# Two Different Template Replicators Coexisting in the Same Protocell: Stochastic Simulation of an Extended Chemoton Model

**DOI:** 10.1371/journal.pone.0021380

**Published:** 2011-07-19

**Authors:** István Zachar, Anna Fedor, Eörs Szathmáry

**Affiliations:** 1 HAS Theoretical Biology and Ecology Research Group, Department of Plant Taxonomy and Ecology, Eötvös University (ELTE), Budapest, Hungary; 2 Department of Plant Taxonomy and Ecology, Eötvös University (ELTE), Budapest, Hungary; 3 Collegium Budapest (Institute for Advanced Study), Budapest, Hungary; 4 Parmenides Foundation, Pullach, Germany; University of Chicago, United States of America

## Abstract

The simulation of complex biochemical systems, consisting of intertwined subsystems, is a challenging task in computational biology. The complex biochemical organization of the cell is effectively modeled by the minimal cell model called *chemoton*, proposed by Gánti. Since the chemoton is a system consisting of a large but fixed number of interacting molecular species, it can effectively be implemented in a process algebra-based language such as the BlenX programming language. The stochastic model behaves comparably to previous continuous deterministic models of the chemoton. Additionally to the well-known chemoton, we also implemented an extended version with two competing template cycles. The new insight from our study is that the coupling of reactions in the chemoton ensures that these templates coexist providing an alternative solution to Eigen's paradox. Our technical innovation involves the introduction of a two-state switch to control cell growth and division, thus providing an example for hybrid methods in BlenX. Further developments to the BlenX language are suggested in the Appendix.

## Introduction

The simulation of complex biochemical systems, consisting of intertwined subsystems, is a challenging task in computational biology. The complex biochemical organization of the cell is effectively modeled by the minimal cell–model called *chemoton*, put forward by Gánti [1], [2], [3], [4]. In this paper we show an application of the process-algebra based programming language BlenX [5], [6], [7] for implementing the chemoton as a stochastic model of a complex chemical system. BlenX was developed for modeling systems whose basic step of computation is a monovalent event, or multivalent interaction between subcomponents [6]. As such it is well suited for modeling elementary or complex chemical reactions [6], Lotka-Volterra type predator-prey dynamics [5] and community dynamics [8].

The language is based on the concept of boxes equipped with binders with π-like processes inside. In the chemical reactions example, boxes represent different molecules, whereas binders express their interaction capabilities and processes handle the manipulation of the binders and drive the internal behavior of the boxes. In classical process calculi, boxes can interact provided that they have identical channel names. BlenX, being a specific type of process calculus itself, has its interactions guided by the compatibility of the binders, which is expressed through an affinity function applied to the type of the binder types [7].

We used the BlenX language to implement the chemoton, and the BetaWB simulator (v2.0.2) to run experiments. The stochastic simulation engine implements an efficient variant of Gillespie algorithms [5] that generates a trajectory of the stochastic evolution of the system: it calculates which reaction will occur next and when it will occur [9]. The key point of the method is to use reaction probability per unit time instead of rate constants. The algorithm used by the BetaWB simulator (called the Next Action Method) uses both Gillespie's Direct method and the First reaction method [9].

The chemoton [1], [2], [3] is an autocatalytic chemical supersystem that satisfies the criteria of life [1], [4]. It consists of three autocatalytic subsystems: a metabolic subsystem (self-reproducing chemical cycle), an informational subsystem (template polymerization cycle) and a boundary subsystem surrounding the former two (membrane). The chemical reactions of the three subsystems are coupled stoichiometrically, which coordinates the growth and division of the cell thus helping the system to be autocatalytic as a whole.

The metabolic subsystem produces the components necessary for its own self-reproduction and those of the other two subsystems. The membrane system provides compartmentalization and keeps the volume of the sphere between certain boundaries whereby it ensures the necessary concentrations which in turn are necessary for the appropriate rate of reactions. The template system controls quantitatively the chemical processes of the whole supersystem [3]. By the introduction of an information carrier template molecule, at least limited heredity [10] can be achieved: when the chemoton reaches a certain size, it splits into daughter spheres, thus it is able to pass on changes in the template molecules to offspring. In the basic model the template consists of only one type of monomer, V, and the only real information the system carries is the distribution of polymers of different lengths. Although information is limited, it is still information.

The number of template variants can be further increased by the addition of another monomer molecule, W. The different monomers can form separate homopolymers an assumption that helps us state the new findings as clearly as possible. Two coexisting templates in a chemoton would be a step toward a larger search space for evolution and toward a qualitative control by the template subsystem over the whole chemoton instead of a quantitative one.

However, internal competition of replicators (in this case templates or polymers) poses a serious problem that arises from the catch 22 of the origins of life: Eigen's paradox. According to the paradox no large genome can be maintained against errors without enzymes, while no enzymes can exist without a large enough genome [11]. One solution proposed the cooperative existence of small template replicators that together may hold enough information (the hypercycle [12]), although it was proven to be insufficient without compartmentation. The stochastic corrector model [13] provides a different solution by enclosing multiple templates (unlinked genes) in compartments. It assumes that replicative templates are competing within reproducing compartments, whose selective values depend on the balance of internal template composition. Stochasticity in replication and compartment fission ensures that the fittest compartment types recur, allowing therefore the stable coexistence of competing replicators. Thus selfish (i.e. faster growing) mutants cannot destroy the system causing the extreme dilution and extinction of slower templates.

We raise the possibility that internal competition may be solved by other means within the chemoton; hence competing templates can coexist within its boundaries. In the chemoton it is the topology of coupling between metabolism and template replication that can maintain coexistence. Our version of the chemoton makes this link explicit by defining reversible reactions linking cell growth with template growth.

**Figure 1:**
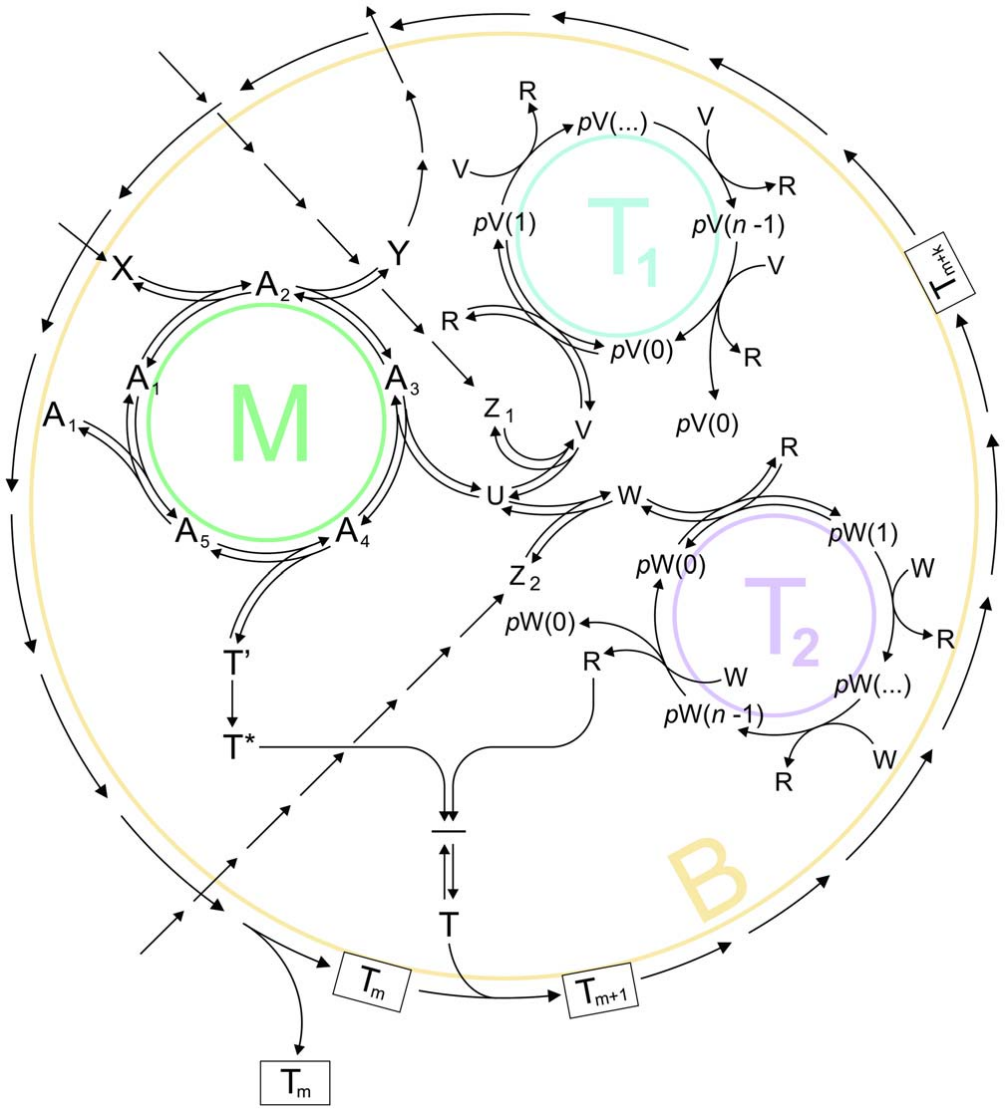
Chemoton with two templates. T_m_…T_m+k_ represent the boundary subsystem, A_1_…A_5_ represent the metabolic subsystem and pV(0)…pV(n−1) and pW(0)…pW(n−1) represent two different template polymerization cycles (informational subsystems), T_1_ and T_2_. Z_1_, Z_2_ and X are food molecules. See text for further details.

In this paper we describe the behavior of two different models. The first model is the standard chemoton model, based on a well-studied set of continuous and deterministic standard nonlinear kinetic differential equations [14]. We show with the help of the BlenX language that a stochastic model behaves in the same way. In the second model (Figure 1) the metabolic cycle produces two different kinds of monomers which form two different kinds of templates in two separate informational subsystems. We show that these two seemingly competing templates (having exponential growth trends *a priori*) can coexist in the system in spite of different polymerization rates.

## Methods

The chemoton is implemented as a series of events, each representing an elementary reaction step (i.e. reversible reactions are split to forward and backward elementary reactions). Components are defined as boxes and are identified by their binder types. Division of the chemoton is initiated and terminated deterministically, but all other processes are stochastic.

The actual growth rate of each component is defined by their kinetic constant *k*, times the amount of the reactants (with appropriate exponents) producing it. The calculated growth rate function is then plugged into the specific event representing the reaction, as the rate of the event. The kinetic factors of a reversible reaction:
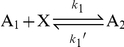
are defined as follows:

let k1 : const = 2.0;

let k1r : const = 0.1;

let rate1 : function = k1 * |A1| * |X|;

let rate1r : function = k1r * |A2|;

where |*n*| indicates the cardinality (amount) of component *n* in the system and k1r in the code equals to *k*_1_′ in the equation. The reactions are called for as events of the following form:

**when**(A1, X :: rate1) **join**(A2);

**when**(A2 :: rate1r) **split**(A1, X);

Since division is a global event that cannot be modeled from molecule to molecule, but only concerning the cell as a whole, we decided to implement it as a non-stochastic process. Division is controlled by a global switch, which starts the growing or the dividing phase of the cell. The standard chemoton reactions are suspended during cell division. Although switching cell states is deterministic and happens instantaneously, the division of amounts is still a stochastic process as will be explained below.

In Gánti's models cell division is triggered automatically when the surface area of the cell doubles. Even if more realistic scenarios exist (for example, in a stochastic model [15] the trigger is the changing osmotic pressure) we chose to use the original assumption for the sake of simplicity. Thus, in our model, cell division always initiates when the surface of the membrane (actually, the amount of T_m_ molecules incorporated into the membrane) reaches a certain, predefined size. At this point cell division starts deterministically by switching the cell from the phase of cell growth to the phase of cell division. (For a smoother splitting mechanism, see [16], where the precise volume is calculated depending on the shape of the cell during division.) The two-state switch (acting as a global signal) is implemented by boxes called Growth and Division:

**when**(Growth : |Tm| = 1000 : inf) **split**(Division, Nil);

**when**(Division : |Tm| = 500 : inf) **split**(Growth, Nil);

The two-state switch is a previously undocumented addition to the BlenX armament of tools. It is a deterministic operator that can be extended to handle more than two states as well. By referring to the state of the switch, multiple events can be triggered immediately. While the referred component (T_m_ here) is properly handled, no infinite loops should occur.

All the stochastic events are conditional on the presence of these signals: the basic chemical reactions of the chemoton are only active when the Growth signal is present; when the Division signal is present all these reactions freeze until cell division is finished.

**when**(A1, X : |Growth|>0 : rate1) **join**(A2);

Conversely, division has been implemented as a set of eliminating reactions which are only active when the Division signal is present. Cell division is modeled by halving all molecular amounts in the system. Only the halving of the amount of T_m_ membrane molecules is deterministic and precise, all other molecules are deleted with their specific deletion-rate until the membrane reaches its post-division size, that is, until T_m_ reaches its lower bound.

**when**(Growth : |Tm| = 1000 : inf) **split**(Division, Nil);

**when**(Tm : |Division|>0 : delrateTm) **delete**(1);

**when**(A1 : |Division|>0 : delrateA1) **delete**(1);

**when**(A2 : |Division|>0 : delrateA2) **delete**(1);

… // delete every other component

**when**(Division : |Tm| = 500 : inf) **split**(Growth, Nil);

where deletion rates are defined as:

**let** factor : **const** = 10.0;

**let** delrateA1 : **function** = factor * |A1|;

**let** delrateA2 : **function** = factor * |A2|;

…

The food molecule X either has a constant concentration, or is constantly added to the system with a specific (fast) rate, which sets the pace for the chemoton, for example:

**let** influx : **const** = 10.0;

…

**when**(X : |X| = 0 : influx) **new**(1);

Template polymerization follows the method of [14]. Initially a double-stranded homopolymer of length *n*/2 is present (it consists of two *n*/2-length polymer strands; *n* monomers in sum). This state of the polymer is called *p*V(0), indicating that zero extra monomer was added to it so far above the basic *n*. During polymerization the double stranded polymer is growing by successively adding extra V molecules (e.g. *p*V(0)+V→*p*V(1)+R) until the total number of V molecules in the polymer reaches 2*n*−1. This state is denoted *p*V(*n*−1). Adding a further V to the polymer, it splits into two *p*V(0) molecules, initiating thus further autocatalytic template cycles. In each of the models discussed here *n* = 6. We entirely ignored the polycondensation threshold value (usually present in chemoton models, e.g. [3], [14], [17]), because it is not necessary in our model for maintaining the growth and division cycles of the chemoton. The mechanism of replication is deliberately kept as a black box: we take a worst-case approach by assuming that replication can result in exponential growth. We are aware of the complication that non-enzymatic replication of nucleic acid templates in general is an unsolved problem [18], but here we address a different issue.

In the double-template model (Figure 1), the template monomers V and W have a common precursor, U. Whether U will be converted to V or W in reversible reactions depends entirely on the availability of food molecules Z_1_ and Z_2_. Since Z_1_ and Z_2_ are represented as entities of constant concentration (assuming that the outside environment is stable and abundant in food), the proportion of V and W (and thus *p*V and *p*W) will be regulated by the kinetic factors of the forward (*k*_V_ and *k*_W_) and backward reactions (*k*_V_′ and *k*_W_′), and the rates of polymerization (*k*_V6_, *k*_V7_, *k*_W6_ and *k*_W7_).

In the simple model, there is only one template polymerization cycle which is directly connected to the metabolic cycle. There is no precursor (U), food molecules Z1 or Z2; V is directly produced by metabolism.

Note that the time scale of simulations is dimensionless; with the proper setting of rates and initial amounts, the behavior scales appropriately with time.

## Results and Discussion

### Basic model

**Figure 2:**
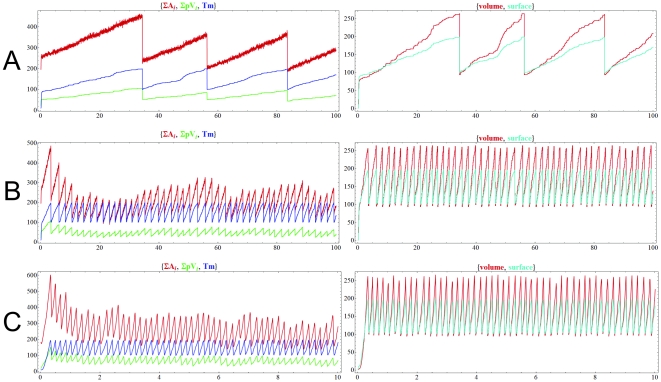
Stochastic behavior of the chemoton. A: The food molecule X has an initial amount of 200 and is constantly added to the system with a low rate (10). B: The influx rate of X is increased (200). C: X has a constant amount (10), representing a stable outside world. Runs were initialized with 200 A_1_, 20 *p*V(0), 10 T_m_ and 1 Growth. Critical T_m_ is at 200. ∑A_i_ stands for the total amount of all metabolites, ∑*p*V_i_ for the total amount of all *p*V polymer stages.

Figure 2 shows the behavior of the basic chemoton in BlenX, with similar kinetic constants as used by [14], [17], [19]. These deterministic models agree that the chemoton maintains stable cell cycles in various conditions. However, in Munteanu and Solé's model [20], small changes in the concentration levels may lead to drastic changes in the replication period. The cause of these differences is still unclear. In the only stochastic model of the chemoton [21] stable cell cycles were found. Our model also confirmed these results.

The run was initialized with 200 A_1_, 20 *p*V(0), 10 T_m_ molecules and 1 Growth signal (and either 200 X and an influx rate, or a constant amount of X). The critical amount of T_m_ was 200: division initiates when the number of T_m_ molecules grows above 200. Right after division, the cell membrane contains 100 T_m_ molecules. The initial concentrations need not be close to the adapted concentrations of the chemoton, as the system self-regulates: each component, independently of their initial amount, reaches its typical value, which is maintained throughout the oscillations. The chemoton can exist stably, with clockwork-like oscillations. Its internal processes are synchronized to the division process, which solely relies on the amount of membrane molecules.

### Double template model

**Figure 3:**
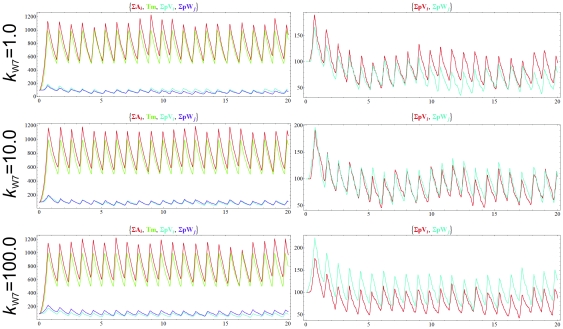
Dynamics of the chemoton with two different templates, *p*V and *p*W, when the concentration of food molecules is constant. *k*_V6_ = *k*_V7_ = *k*_W6_ = 1, critical T_m_ = 1000. Top row: the polymerization rate of V (*k*_V7_) and W (*k*_W7_) are identical (1). Middle row: *k*_W7_ = 10. Bottom row: *k*_W7_ = 100. In the last two cases, *k*_V7_ = 1. Volume and surface variables are omitted from the figure. Initial amounts: 100 A_1_, 100 *p*V(0), 100 *p*W(0), 100 T_m_ and 1 Growth. X has constant amount at 20, Z_1_ and Z_2_ at 10. Note that since division is set to a 100 times slower than in Figures 2 and 4, removal of molecules is actually slower than growth. This has no effect on the outcome of the simulation, as the removal process is deterministic. ∑A*_i_* stands for the total amount of all metabolites, ∑*p*V*_i_* and ∑*p*W*_j_* for the total amount of all *p*V and *p*W polymer stages, respectively.

Figure 3 shows now a second template subsystem can coexist with the first one. We investigated the effect of different polymerization rates on this model. In Figure 3 top row the two templates have identical polymerization rates, and both V and W are created in reactions with identical kinetic constants. As expected, the two templates stably coexist.

In the second experiment, one of the polymers has a higher polymerization rate. Usually, if an autocatalytic entity has a higher growth rate than another one, its amount increases (due to the autocatalytic nature of the template) to a point where the other template is practically diluted to extinction. On the contrary, in the chemoton two homopolymers can stably coexist, even if they have different individual growth rates. Figure 3 middle and bottom rows show that templates stably coexist when *p*W has a polymerization rate 10 or even 100 times higher than that of *p*V, respectively. In the chemoton all subsystems are stoichiometrically coupled, meaning that the growth of each component is synchronized with the overall growth of the chemoton.

**Figure 4:**
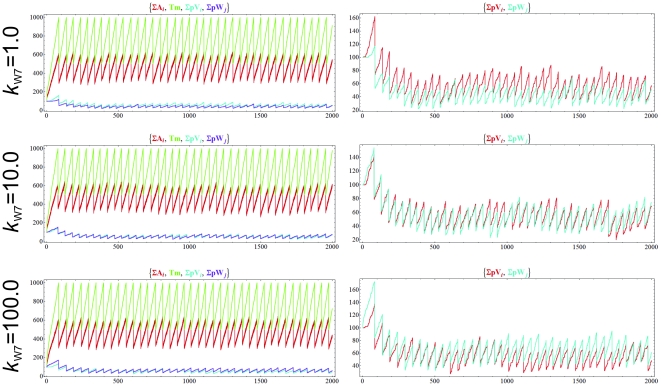
Dynamics of the chemoton with two different templates, *p*V and *p*W, when the main food molecule (X) has a low influx rate. *k*_V6_ = *k*_V7_ = *k*_W6_ = 1, critical T_m_ = 1000. Top row: the polymerization rate of V (*k*_V7_) and W (*k*_W7_) are identical. Middle row: k_W7_ = 10. Bottom row: *k*_W7_ = 100. In the last two cases, *k_V7_* = 1. Volume and surface variables are omitted from the figure. Initial amounts: 100 A_1_, 100 X, 100 *p*V(0), 100 *p*W(0), 100 T_m_ and 1 Growth. Influx rate of X is 10; Z_1_ and Z_2_ are still constant.

We also tested what happens if the external source of X is not constant, but has a low influx rate (Figure 4). This introduces both extra stochasticity, and a reduced pace for the oscillatory cycles (note time scale), but still the experiments yield similar results.

Although the whole template subsystem is still stoichiometrically coupled with metabolism and membrane growth the two templates have internal dynamics. These are independent of the dynamics of the chemoton as a whole: the total amount of *p*V(0) and *p*W(0) polymers is the same as the amount of *p*V(0) in case of the basic model. The internal template-competition is the consequence of the OR type branching of the reactions of U (U+Z_1_→V or U+Z_2_→W). The reversible nature of the reactions producing V and W and the periodic fission of the chemoton ensures that the faster template cannot wipe out the slower one.

**Figure 5:**
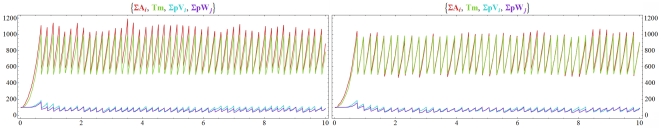
Comparison of different metabolic subsystems. The chemoton on the left consists of a 5-member metabolic cycle (A_1_ to A_5_), while the chemoton on the right harbors a 12-strong metabolism (A_1_ to A_12_). The extra metabolites feed on X and the previous metabolite, and produce the next metabolite in the cycle. The larger number of metabolic partners slightly decreases the total amount of metabolites, ∑A*_i_*. This is a phenomenon that is supported directly by the numerical results of deterministic models: the larger the number of intermediates in the metabolic cycle the less the total amount of metabolic molecules is in a splitting equilibrium. It is a consequence of the relative position where T_m_ is produced in the cycle: the earlier it is generated (i.e. the more metabolites are in the cycle after T_m_ is generated), the less the total amount of metabolites will be, as T_m_ defines the critical value for splitting.

To test the robustness of the chemoton, we have investigated different metabolic subsystems as well. Figure 5 compares the behavior of the model with *m* = 12 metabolites with *m* = 5 metabolites: the difference is almost undetectable, indicating that the model is capable of handling larger autocatalytic cycles as well. Smaller metabolic subsystems can be crafted, though somewhat forcibly: Gánti has defined the metabolic subsystem of five members as the minimal cycle consisting of elementary reaction steps. Fewer metabolites means that some of the elementary reaction steps should be combined, yielding thus non-elementary reactions. This, having no effect on the simulation at all, blurs the clarity of the original chemoton model, thus we mention it only briefly that results for *m* = 3 are almost identical to those for *m* = 5.

### Conclusions

We have shown in this paper how to implement a stochastic version of the chemoton with the help of the BlenX programming language. BlenX was developed for implementing systems that can be built up by the basic interactions of components; thus, chemical reactions are quite straightforward to implement in this language. Since the chemoton is a theoretically important and well-studied system, this study could be a milestone for BlenX applications.

A specific, somewhat contradictory solution was the introduction of a deterministic two-state switch in our BlenX code. It might seem strange to introduce a deterministic process in a stochastic model; however, it was very important for controlling the timing of the different stages of the cell cycle, namely, the growth and the division of the chemoton, being thus a prime example of hybrid methods available in BlenX.

The new theoretical result of our model is that two competing templates can coexist in the chemoton thanks to the topology of the coupling of its chemical reactions. This finding suggests that the constraint on coexistence of different templates, as assumed in the formulation of Eigen's paradox, may generally be more relaxed than previously thought. Results are robust for both polymer-size and the size of the metabolic subsystem.

### Notes and suggestions for BlenX

The most important next step would be to allow the chemoton to host a large set of possibly interacting templates, yielding heteropolymers as well. This would open the scene for novel combinations and also for mutations (i.e. hereditary variations) to appear. By this way the evolution of templates could be introduced into the model. However, evolution cannot be directly modeled in BlenX. Evolution requires the random generation of sufficient variability but at present, there is no method or function in BlenX that could generate random variation (we are aware of the fact that this is work in progress). At present, the only way to work around is to manipulate the results of successive BlenX simulations by external scripts [22]. A random number generator therefore should be integrated with box/process/interface creation, to allow the generation of new, programmable boxes on the fly, or the mutation of existing boxes, processes, binder types, and binder affinities during runtime.

This also requires the referencing of boxes or processes that were not declared prior to running. An alternate naming system could rely on parental relations rather than structural congruence: each entity could be tracked, even if their internal structure is unknown, by their parent entities. We conclude that the BlenX language is a very good medium to simulate closed systems, with predefined actors, such as predator-prey dynamics, or chemical reactions. However, as evolutionary biologists, we would like to see a more convenient way to model evolutionary systems as well. The addition of the random number generator, its integration with process-generation, and the possible referencing of undeclared entities would open the door for evolutionary modeling in BlenX, and possibly would give a boost for the productivity of the language by making it a very useful tool in a number of fields.

Provided that these improvements are present in a future release of BlenX, real evolutionary simulations will become feasible with the existing powerful capabilities of the language. Simulating more advanced chemoton models in a stochastic way in BlenX would yield important insights about the coexistence of replicators in compartments, something which has been a holy grail for researchers of prebiotic evolution for decades.

## References

[1] Gánti T (1971).

[2] Gánti T (1978). Chemical systems and supersystems III. Models of self-reproducing chemical supersystems: the chemotons.. Acta Chim Acad Sci Hung.

[3] Gánti T (2003).

[4] Gánti T (2003). The Principles of Life.

[5] Dematté L, Priami C, Romanel A (2007). BetaWB: Modelling and simulating biological processes; San Diego, CA, USA.

[6] Dematté L, Priami C, Romanel A (2008). The BlenX language: A tutorial.

[7] Ciocchetta F, Priami C (2008). The BlenX language with biological transactions..

[8] Livi CM, Jordán F, Lecca P, Okey TA (2011). Identifying key species in ecosystems with stochastic sensitivity analysis.. Ecological Modelling.

[9] Gillespie DT (1977). Exact stochastic simulation of coupled chemical reactions.. The Journal of Physical Chemistry.

[10] Szathmáry E, Maynard Smith J (1993). The Origin of Genetic Systems.. Abstracta Botanica.

[11] Eigen M (1971). Self-organization of matter and the evolution of biological macromolecules.. Naturwissenschaften.

[12] Eigen M, Schuster P (1978). The Hypercycle. A Principle of Natural Self-Organization. Part C: The Realistic Hypercycle.. Naturwissenschaften.

[13] Szathmáry E, Demeter L (1987). Group Selection of Early Replicators and the origin of Life.. Journal of Theoretical Biology.

[14] Fernando C, di Paolo E, Pollack J, Bedau M, Husbands P, T I, Watson R (2004). The chemoton: A model for the origin of long RNA templates..

[15] Mavelli F, Ruiz-Mirazo K (2007). Stochastic simulations of minimal self-reproducing cellular systems.. Philosophical Transactions of the Royal Society B: Biological Sciences.

[16] Carletti T, Fanelli D (2007). From chemical reactions to evolution: Emergence of species.. EPL.

[17] Csendes T (1984). A simulation study on the chemoton.. Kybernetes.

[18] Fernando C, Kiedrowski GV, Szathmáry E (2007). A stochastic model of nonenzymatic nucleic acid replication: “Elongators” sequester replicators.. Journal of Molecular Evolution.

[19] Fernando C (2005).

[20] Munteanu A, Solé RV (2006). Phenotypic diversity and chaos in a minimal cell model.. Journal of Theoretical Biology.

[21] Segbroeck SV, Nowe A, Lenaerts T (2009). Stochastic Simulation of the Chemoton.. Artificial Life.

[22] Dematté L, Priami C, Romanel A, Soyer O (2008). Evolving BlenX programs to simulate the evolution of biological networks.. Theor Comput Sci.

